# Draft Heidelberg Collaboration Statement of Purpose (22/9/2000)

**DOI:** 10.2196/jmir.2.suppl2.e11

**Published:** 2000-09-22

*Warning - this is an unedited draft, which will be replaced by a final version mid of October [will be posted at this URL].*

Following a meeting convened to discuss the EU-funded MedCERTAIN project on improving information quality on the Internet, a broad group of individuals from 4 continents and 21 countries, drawn from a wide range of sectors including industry, leading Web sites, governmental and intergovernmental bodies, academia, consumer organisations, standards and ethical code bodies and publishers agree that:

1. Despite the enormous value for the public, patients and professionals of the health and medical Internet, the participants are concerned about its potential for harm. However, it is also important not to limit freedom of expression and use of the internet for health related purposes.

2. The participants wish to explore in an ongoing collaborative effort opportunities for helping people, patients and professionals to identify health information useful to them, addressing certain key issues, including:

- What is an appropriate way to describe the characteristics of the full range of Internet health and medical sites ?
- How can the descriptions be summarised as metadata for different purposes and users of health information ?
- How do the identity, training and other factors (eg. self reporting vs. external audit) influence the description and summary ?
- Can criteria be developed to indicate quality for different users and purposes ?
- How can the potential of information and communications technology, such as the MedCERTAIN project, be harnessed in a consistent and cost-effective way ?
- What are the legal and other implications of trustmarks and other devices or mechanisms to denote quality ?
- How to establish and motivate a worldwide, representative network of evaluators?
- What are the areas of potential overlap with other initiatives and organisations?

3. The participants resolve to work together, building on experience and expertise of this group, key quality improvement initiatives such as Discern, eHealth ethics code, HONcode, Hi-Ethics code, OMNI and others, and working towards a methodological clarification and resolution of the above and other related issues in the near future, providing constructive support to the MedCERTAIN and other projects and continuing to build together trust on the health Internet.

*Participants:*

Lucas M. Bachmann (Horten Zentrum für praxisorientierte Forschung und Wissenstransfer, Switzerland), Carl R. Blesius (MedCERTAIN, Germany), Markus Blume (mymedia GmbH, Germany), Carl J. Brandt (NetDoktor.com, Denmark), Dan Brickley (MedCERTAIN, United Kingdom), Alejandro Cacherosky (Buenos Aires Health Secretariat, Argentina), Ken Campell (Leukaemia Research Fund, United Kingdom), Richard Cleland (Federal Trade Comission, U.S.A.), Phil Cross (MedCERTAIN, United Kingdom), Elenice de Castro (PAHO/WHO Pan American Health Organization / World Health Organization, Brazil), Guy de Roy (Ordre des Medecins Conseil National, Belgium), Tony Delamothe (BMJ, United Kingdom), Martin D. Denz (Swiss Medical Informatics Association / Universitätsspital Zürich, Switzerland), Persephone Doupi (Erasmus University, Netherlands), Joan Dzenowagis (WHO World Health Organization, Switzerland), Christer Edling (The Swedish Society of Medicine, Sweden), Gunther Eysenbach (University of Heidelberg / MedCERTAIN, Germany), Gerard Freriks (TNO, Netherlands), Franz Frühwald (Internationales Büro der Österreichischen Ärztekammer, Austria), Lisa Gray (BIOME, United Kingdom), Pelle Gustafsson (Swedish Medical Association, Sweden), Gerhard Heine (European Commission, Luxembourg), Katrin Hörner (Arztpartner / Almeda, Germany), Robert Hsiung (University of Chicago, U.S.A.), Jostein Ingulfsen (Norwegian Board of Health, Norway), Thomas Isenberg (German Association of Consumer Organisations - Arbeitsgemeinschaft der Verbraucherverbände, Germany), Edward Jacob (KNMG, Netherlands), Alex R. Jadad (Canadian Cochrane Centre, Canada), Jacobo Kelber (ensalud.net, Mexico), Hugo Kitzinger (mymedia GmbH, Germany), Inge Kokot (Deutsches Grünes Kreuz e.V., Germany), Hans-Joachim Koubenec (Stiftung Warentest, Germany), Michel Labrecque (Université Laval Québec, Canada, Canada), Kristian Lampe (Finnish Office for Health Technology Assessment / MedCERTAIN, Finland), Stephane Lejeune (Swiss Cancer League, Switzerland), Leonard B. Lerer (INSEAD, France), Odile Leroy (PasteurMed, France), Nicolas Lienert (Medgate AG, Switzerland), Pål Lindström (LocusMedicus, Sweden), Sándor Lipp (Hungarian Ministry of Health, Hungary), Leena Lodenius (Finnish Duodecim Medical Society, Finland), Antti Malmivaara (Finnish Institute of Occupational Health, Finland), Miquel Angela Mayer Pujadas (Official Medical College of Barcelona, Spain), Peter Mills (EncycloMedica Ltd, United Kingdom), Cesar Molinero (Planet Medica, Belgium), Marc Muret (Zürcher Aerzte für Klassische Homöopathie, Switzerland), Wolfgang Nagel (GesundheitScout24 GmbH, Germany), Tim Nater (Health on the Net Foundation HON, Switzerland), Joerg Nitzsche (Zentralbibiliothek für Medizin Koeln, Germany), Debra O´Connor (La Trobe University, Australia), Gert Purkert (Arztpartner / Almeda, Germany), Ramon Sarrias Ramis (Official Medical College of Barcelona, Spain), Christine Reuter (A MedWorld AG, Germany), Ahmad Risk (Internet Healthcare Coalition, United Kingdom), Carl Bénédict Roth (GetWellness, Switzerland), Sebastian Schmid (Arztpartner / Almeda, Germany), Christiane Schmitz (Heinrich Heine Universität, Germany), Luk Schoonbaert (Belgacom Multimedia Ventures, Belgium), Frank Schuler (gfp Kommunikation Köln, Germany), Ulrich Schwanke (DocCheck, Germany), Sasha Shepperd (Imperial College School of Medicine, United Kingdom), Myra Sidrassi (MedCERTAIN, Germany), Chris Sigouin (McMaster University, Canada), Chris Silagy (Monash Medical Centre, Australia), Denise Silber (Internet Healthcare Coalition, U.S.A), Martin Sonderegger (Universitätsspital Zürich, Switzerland), Anke Steckelberg (Universität Hamburg, Germany), Frederik Tautz (Heinrich Heine Universität, Germany), Nicolas P. Terry (Center for Health Law Studies, St. Louis University, U.S.A.), Christian Thomeczek (Ärztliche Zentralstelle Qualitätssicherung, Germany), Wouter Tukker, (Praha Communications, Czech Republic), Gerard H. van der Zanden (The Netherlands Institute for Health Promotion and Disease Prevention NIGZ, Netherlands), Georg von Below (Swiss Medical Assoc./FMH, Switzerland), Frank von Danwitz (Deutsches Diabetes-Forschungsinstitut, Germany), C.-Peter Waegemann (Medical Records Institute, U.S.A.), Thomas Wetter (Dept. Of Medical Informatics, Universität Heidelberg, Germany), Petra Wilson, (European Commission, Belgium), Margaret A. Winker (JAMA, U.S.A.), Jeremy Wyatt (University College London, United Kingdom), Gabriel Yihune (MedCERTAIN, Germany)

